# Formulation and *In-vitro* Evaluation of pH-Sensitive Oil Entrapped Polymeric Blend Amoxicillin Beads for the Eradication of *Helicobacter pylori*

**Published:** 2012

**Authors:** Girish Kumar Tripathi, Satyawan Singh, Gopal Nath

**Affiliations:** a *Industrial Pharmaceutic Laboratory, Saroj Institute of Technology and Management Lucknow-226001, India.*; b *Department of Microbiology and Infectious Disease, Banaras Hindu University Varanasi, India*.

**Keywords:** Amoxicillin, pH sensitive drug delivery system, Minimum inhibitory concentration (MIC), Gastric retention

## Abstract

Oral pH sensitive drug delivery systems are of utmost importance as these systems deliver the drug at specific part of the gastrointestine (GI) as per the pH of GI, resulting in improved patient therapeutic efficacy and compliance. The pH range of fluids in various segments of the GI tract may provide environmental stimuli for drug release. The aim of this study was to design buoyant beads containing amoxicillin (Am) and to evaluate its potential for the eradication of *Helicobacter* *pylori (H. pylori)*. The gel bead of gellan, wherein the oil was entrapped, was blended with hydroxypropyl methyl cellulose or Carbopol 934. Buoyant beads of gellan were prepared through ionotropic gellation technique to achieve the controlled and pH-sensitive drug release in stomach. The effects of processing variables such as particle size, buoyancy, percent encapsulation efficiency and *in-vitro* antimicrobial activity were evaluated. The scanning electron micrograph indicated that prepared beads were spherical in shape and all the beads showed satisfactory floating efficiency in the phthalate buffer solution. The diameter of the gel beads was increased through raising the gellan gum and calcium carbonate concentration. The formulation exhibited sustained release profile and was best fitted in the Peppas model with n < 0.45. Subsequent coating of microbeads exhibited zero-order sustained pattern of the drug release up to 8 h. *In-vitro* growth inhibition study showed complete eradication of the isolated *H. pylori *strain .These results provide evidence that the optimized formulation bearing antibiotics like amoxicillin should be useful in *H. pylori *treatment.

## Introduction

Oral gastro-retentive delivery system exhibits a prolonged controlled release which has been a topic of interest in terms of their potential for targeted release in the stomach. These systems are also appropriate for drugs which are locally active in gastric mucosa of the stomach, in particular case of antibiotic administration for *H. pylori *eradication to treat the peptic ulcer and other associated diseases (1).

**Figure 1:**
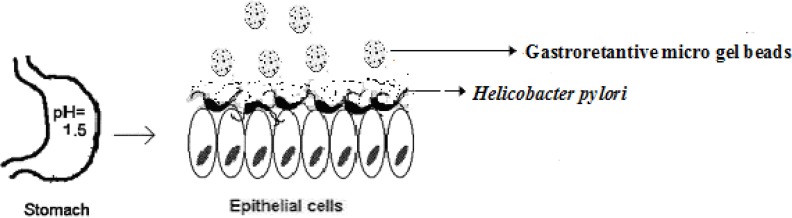
Schematics of targeted drug delivery approach for *H. pylori *eradication located within the stomach (3).

*H. pylori *are small, spiral, microaerophilic, Gram-negative bacteria and it is recognized to be associated with gastritis and duodenal ulcers. The microorganism has also been reported to be involved with the pathogenesis of other diseases, such as chronic atrophic gastritis, adenocarcinoma of the body or antrum of the stomach, gastro-esophageal reflux disease, peptic esophagitis *etc.* (2). The bacteria penetrates the gastric mucus layer and fixes itself to various phospholipids and glycolipids in the mucus gel. Therefore, the access of antimicrobial drugs to the site is restricted from both the lumen of the stomach and the gastric blood supply. Conventional tablets or capsules are, in general, used for eradication therapy, but these preparations do not remain in the stomach for long duration. Therefore, it is difficult to reach the minimum inhibitory concentrations (MIC) in the gastric mucus where *H. pylori *colonize. It is therefore necessary to design the drug delivery systems that not only can alleviate the shortcomings of conventional delivery but can also deliver antibiotics to the infected cell lines (Figure 1).

The stomach specific gastroretantive (GR) and controlled release delivery system can maintain the MIC of an antibiotic in the gastric region where *H. pylori *exist and thereby improve the therapeutic efficacy. The absorption of an antimicrobial agent from the gastric lumen into the mucus through the mucus layer is believed to be more effective for *H. pylori *eradication than absorption through the basolateral membrane (from blood).

Narkar *et al*. 2010 (4) prepared and evaluated stomach-specific controlled release mucoadhesive drug delivery system containing amoxicillin trihydrate. Rajinikanth and Mishra (5) reported gellan gum-based floating beads. Babu *et al*. 2010 (6) prepared controlled release gellan gum macro beads of amoxicillin.

It is therefore necessary to design drug delivery systems that not only can alleviate the shortcomings of conventional delivery vehicles but can also deliver the antimicrobial agent to the infected cell lines. Design of novel mucoadhesive, a pH-sensitive formulation, may improve the local therapy of GI and minimize the premature drug degradation. Stomach-specific antibiotic drug delivery, for instance, would be highly beneficial in the treatment of *H. pylori *infection in peptic ulcer disease (7-9). Amoxicillin is one of the most active and predictable antimicrobial agents against *H. pylori *and also has rapid bactericidal activity compared with various antibiotics and antiulcer agents (10, 11). Amoxicillin inhibits transpeptidase and stops the synthesis of peptidoglycan, thereby weakening the cell wall and making *H. pylori *more susceptible to death due to bursting.

There are several benefits in controlled drug delivery systems over the conventional pharmaceutical dosage form: reduction in drug plasma level and fluctuation, reduction in adverse side effect and improvement in tolerability, patient comfort, complains and finally reduction in healthcare cost (12-14). Gellan is an anionic, high molecular weight, deacetylated exocellular polysaccharide gum produced as a fermentation product through a pure culture of *Pseudomonas elodea* (15). In the present study, oil entrapped buoyant gel bead of Amoxicillin consisting of hydrophilic polymer (carbopol 934P or hydroxylpropyl methyl cellulose), blended with gellan gum, was designed, using calcium carbonate (CaCO_3_) as a gas forming agent. The primary objective of the work was to develop a consistent formulation of Amoxicillin that enjoys all the advantages of a floating single unit dosage form, but at the same time, it lacked disadvantages of single unit dosage forms, like sticking or being obstructed in GI. The release behaviour of the gel beads capable of floating in gastric fluid was investigated with the aim of achieving a GR, multiple units, and controlled release formulation of amoxicillin.

**Table 1:** Composition for amoxicillin loaded polymeric blended gellan gum beads

**Formulation**		**Drug** **(% w/v)**	**Gum** **(% w/v)**	**oil** **(% w/v)**	**Calcium carbonate** **( %w/v)**
**(COB)**	**(HOB)**				
K_1_	S_1 _	0.62	1.5:1.0	05	0.55
K_2_	S_2_	0.62	1.5:1.0	10	0.55
K_3_	S_3_	0.62	1.5:1.0	15	0.55
K_4_	S_4_	0.62	1.5:1.0	05	1.50
K_5_	S_5_	0.62	2.0:0.5	05	0.55
K_6_	S_6_	0.62	2.0:0.5	10	0.55
K_7_	S_7_	0.62	2.0:0.5	15	0.55
K_8_	S_8_	0.62	2.0: 0.5	05	1.50

COB = gellan: carbopol blend formulation. HOB = gellan: HPMC blend formulation. Gum** = **gellan: carbopol /HPMC. Oil** = **mineral oil

## Experimental

Amoxicillin was obtained as a gift sample from Ranbaxy Laboratories, Gurgoan, India. Gellan gum was purchased from Sigma-Aldrich Chemicals Ltd, New Delhi, India. Carbopol 934P and hydroxypropyl methylcellulose K4M were obtained as gift samples from Ranboxy laboratory Devash, India. Calcium chloride and ethyl cellulose were obtained from SD. Fine Chem. India. Light mineral oil was obtained from the Central Drug House, India. All other ingredients, reagents and solvents were of analytical grades. Brucella broth and fetal calf serum were purchased from Himedia Mumbai, India.

*Preparation of polymeric blend oil entrapped bead*

Oil entrapped polymeric blend gel bead of amoxicillin was prepared through the ionic gelation method. Aqueous solution of gellan gum (1.5-2.0 % w/v) was prepared using de-ionized water and heating at 70°C. The gellan gum solution below 35°C was successively dispersed into the slurry of 0.5 -1.0 % w/v of HPMC or carbopol 934P in order to prepare specific ratio of blended polymeric dispersion (Table 1) with continuous stirring for 20 min. The drug (0.62 % w/v) and calcium carbonate (0.55-1.5 % w/v) were dispersed uniformly into 20 mL of the polymeric blended mixture with continuous stirring until a uniform dispersion was obtained. The mixture was emulsified with 05-15 % w/v of light mineral oil using Silverson emulsifier (Hicon, L5M-4, India) with continuous stirring at 500 rpm for 5 min. The resulting drug of the loaded emulsions was added through a 21G syringe needle into 100 mL of 0.45 mol/mL of calcium chloride (CaCl_2_) solution. After 5 min of curing, the gel beads of amoxicillin loaded gellan gum blended, either with hydroxypropyl methylcellulose (HOB) or carbopol 934P (COB). Gel beads took place after 5 min of curing, the formed beads were washed with distilled water, collected and dried at 40°C for 6 h in tray dryer.

*Coating of gel beads*

The formulated microgel beads were selected for the optimization in order to modify the drug release pattern further. The coating parameters were 5-10% (w/v) ethylcellulose (EC) solution in acetone and coating time was fixed (5-10 min). Gel beads (2 g) were placed in a fluidized bed dryer (TG 100, Retsch, Germany) and the coating solution was sprayed on the fluidized beads using a spray gun for a period of 10 min at an air inlet speed of 220 m/s at room temperature. The beads were dried at room temperature for a period of 24 h until all solvents were evaporated, leaving a film of EC coat on the gel beads (Table 2).

**Table 2:** Independent variables of the formulation bead coated with ethyl cellulose

**Formulation**	**EC concentration** **(code)**	**Time of coating** **%w/v**	**%drug release** **(min)**	**R** ^2^ **t**_480 (min.)_
K_41_	5	5	88 ± 1.3	0.982
K_42_	5	10	85 ± 1.4	0.975
K_43_	10	5	80 ± 1.6	0.934
K_44_	10	10	79 ± 1.4	0.926

EC: ethyl cellulose; R^2^: correlation coefficient, derived from zero order kinetics of the coated formulation; K_41_, K_42_, K_43_ and K_44 _: ethylcellulose coated optimized formulation of the batch K_4;_ A: Mean ± SD (n = 3).

*Morphology and size*

The external and internal morphology of micro gel beads were studied through scanning the electron microscopy (SEM). Particle size of the prepared beads were determined in three sets using an optical microscope (Model BH-2, Olympus, Japan) fitted with a stage and an ocular micrometer. Mean diameter was calculated through measuring the diameter of 20 dried beads.

*In-vitro floating study*

*In-vitro* floating study was performed using a USP 24 dissolution apparatus II containing 900 ML of phthalate buffer solution (pH = 3.4). The medium temperature was kept at 37 ± 0.5°C. The floating beads (1.0 g beads) were soaked in the dissolution medium and the medium was agitated with a paddle at 50 rpm. After the agitation, the beads that were floated on the surface of the medium and those that settled down at the bottom of flask were recovered separately. The percentage of floating was measured through the visual observation (16).

*Determination of encapsulation efficiency and drug loading*

Accurately weighed (100 mg) grounded powder of beads was soaked in 100 mL phosphate buffer (pH = 7.5) and allowed to be disintegrated completely for 4 h. The resulting dispersion was sonicated using a probe sonicator (UP 400 s, Dr. Hielscher GmbH, Germany) for 30 min and then filtered through a 0.45 μm filter. The polymeric debris was washed twice with fresh phosphate buffer to extract any adhered drug. The drug content was determined spectrophotometrically (Systronics, Mumbai, India) at 334.5 nm against constructed a calibration curve. The drug content (DC) was calculated according to Equation 1.

(DC) = (amount of drug in beads / amount of beads) × 100 (Equation 1)

The encapsulation efficiency (EE) was calculated by Equation 2.

EE (%) = (C D) / (T D) × 100 (Equation 2)

Here, C D is the calculated drug content and T D is the theoretical drug content.

*In-vitro drug release*

*In-vitro* dissolution studies were performed for all the formulation gel beads using USP 24 dissolution test apparatus II with a basket type (17). An accurately weighed 50 mg amount of the beads were taken into 900 mL dissolution medium of the simulated gastric fluid (fasting state condition, pH = 1.2) or phthalate buffer solution (fed state condition, pH = 3.4) conditions maintained at a temperature of 37 ± 0.5ºC and stirred at a speed of 50 rpm. Sample aliquots (10 mL) were withdrawn at 0.5, 1.0, 2.0, 3.0, 4.0, 5.0, 6.0, 7.0 and 8.0 h and the volume was replaced with an equivalent amount of a plain dissolution medium. The collected samples were filtered and suitably diluted and analyzed at 334.5 nm using a UV-visible spectrophotometer. Additionally, an experimental batch of BE and BF containing 10 mg Amoxicillin and lactose (q.s.) filled in a capsule (#2) was used as a reference formulation.

*In-vitro growth inhibition studies*

The bacterial strain used in this study was originally isolated with gastric biopsy from the peasant suffering from chronic gastritis and peptic ulcer in Institute of Medical Science, Banaras Hindu University Varanasi-India. Turbid metric method with slight modification was employed to evaluate the growth inhibition (18). The protocol of the study was approved by Institutional Animals Ethical Committee of the BHU. To suppress the growth of indigenous or exogenous contaminating bacteria, the isolated biopsy sample was grown in brucellla agar (Merck Co, Germany) containing 10% horse blood, Vancomycin, polymyxin B and amphotericin and incubated at 37°C for 7 days. Isolated sample was subcultured on Brucella agar containing 10% horse blood without antibiotics and incubated at 37°C for three days in microairofilic condition.

*H. pylori *strains were grown in brucella broth at 37°C after 7 days in microaerobic atmosphere (5% O2, 10% CO_2_, 85% N_2_). The growth of the bacteria was monitored by measuring the optical density (OD) of broth cultures with spectrophotometrically at *λmax* of 640 nm (19). The numbers of bacteria were determined in terms of optical density at *λmax of *640 nm with one optical density unit corresponding to 10^8 ^colony-forming units (CFU)/mL. To study the effect of formulations on *H. pylori *growth inhibition, 10 mL of nutrient broth containing *H.pylori *were transferred into sterile test tubes. Plain drug (Am) and optimized formulation of the ethylcellulose coated batches were taken containing amoxicillin equivalent to 32 µg/mL which is twice in concentration with respect to MIC (16 µg/mL) and added to the tubes and all the tubes were incubated at 37°C in a microaerobic atmosphere for 24 h. The tubes containing culture were shaken at 100 rpm at 37°C in a microaerobic atmosphere condition in incubator for 24 h. Then, 100 μL of nutrient broth of *H.pylori *containing drug and different formulations were removed at various time points (4, 8 and 12 h) and optical density was determined to assess the growth inhibition of bacteria through counting the viable colony using spectrophotometer. The percentage of growth inhibition was calculated using Equation 3.

$\mathrm{\% Growth inhibition}=\frac{{\mathrm{OD}}_{\mathrm{TP}}-{\mathrm{OD}}_{\mathrm{TS}}}{\mathrm{Volume of Saline Load}}\times 100$ (Equation 3)

Here, OD_TP _= Optical density of test organism at particular interval. OD_TS_ = optical density of test mixture at same time interval.

*Kinetic release evaluation*

To investigate the mode of drug release from the micro gel bead, the release data were analyzed with various release kinetic models (zero order, higuchi and korshmaer-Peppas) were applied to elucidate the mechanism of drug release from the beads in the fed state (20-22). These Kinetic models were used to analyse the dissolution study, , Equations 4, 5 and 6.

Zero-order model 

M_t_ = M_0_ + K_0_t (Equation 4)

Higuchi model 

M_t_ = M_0_ + K_H _t^0:5^ (Equation 5)

Korshmaer-Peppas model

M _t_ /M ∞ = k (t)^ n^ (Equation 6)

Here, Mt is the amount of drug dissolved in time, M_0_ is the initial amount of drug, K_0_ is the zero order release of constant and K_H_ is the Higuchi rate constant. M_t_/M∞ is the fraction of drug release at time t, k is the release rate constant, and n is the release exponent indicative of the mechanism of release.

*Statistical analyses*

The experimental results are expressed as mean ± SD (standard deviation). Statistical evaluation of data was performed using an analysis of variance (ANOVA), depending on the outcome of the ANOVA (Dunnett’s multiple comparison test). The evaluation data was used to assess the significance of differences. Statistically significant differences between the means of batches were defined as p < 0.05.

## Results and Discussion

The hydrophilic polymer was blended in gellan gum, in optimized ratio, to enhance the mucoadhesion efficacy of prepared microgel beads. Mucoadhesion involves different kinds of interaction forces between mucoadhesive materials and the mucus surface; these include electrostatic attraction, hydrogen bonding, Vander Waals forces, mechanical interpenetration *etc*. A combination approach, *i.e.* mucoadhesive and pH-sensitive controlled delivery system, is explored for the effective and improved treatment of *H. pylori *infection.

**Figure 2:**
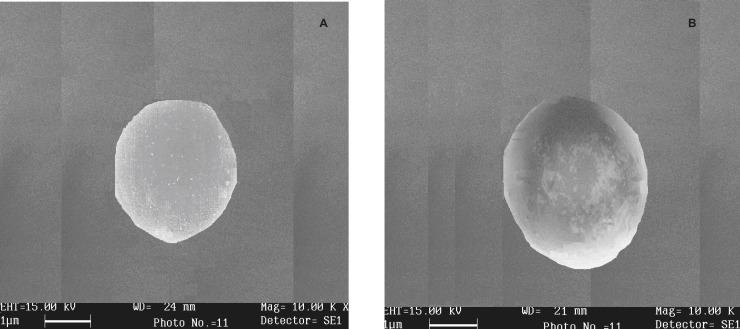
Scanning the electron micrograph of dried beads of (A) batch K_4_(COB formulation) and (B) batch S_5 _(HOB formulation)

*Morphology and size*

The shape and surface morphology of prepared microgel beads were investigated using scanning electron microscopy. SEM of Amoxicillin-loaded oil entrapped gellan gum blended with carbopol 934P of batch K_4_ (COB formulation) and HPMC blended batch S_5_ (HOB formulation) are shown in Figure 2. Batch K_4_ gel beads were white, translucent and rigid, whereas, batch S_5_ gel beads were off-white, translucent and elastic. The beads size of formulation COB varied between 1.85 ± 1.3 and 2.28 ± 1.3 mm while that of COB was between 1.34 ± 1.4 and 1.62 ± 1.6 mm (Table 3). The diameter of microbeads were increased significantly (p < 0.05) as gellan gum or CaCO_3 _concentration were increased. Similar findings have been observed by Rajinikanth *et al.* 2009 (5).

*In-vitro floating study*

The floating ability of COB formulation was in the range of 61 ± 0.4 % to 85 ± 0.4 % and formulation HOB varied from 60 ± 0.4 % to 79 ± 0.6 % (Table 3). By increasing CaCO_3_ concentration, floating ability of micro gel beads was increased. This could be attributed to the increase in amount of calcium ions (Ca^+2^) and consequently the amount of evolved carbon dioxide (CO_2_) is entrapped in the gel network of the formulation cause gel rise to the surface of the dissolution medium.

*Determination of* *encapsulation efficiency and drug loading*

The parameters of the formulation effect on encapsulation efficiency and drug content are shown in Table 3. Encapsulation efficiency of the drug was found to be consistently higher in the prepared formulation of COB (79 ± 1.7 – 96 ± 1.8 %) and HOB (73 ± 1.5 to 88 ± 1.6 %). It has been observed that the encapsulation efficiency of beads is increased through increasing the gellan concentration; the result was similar as reported by Narkar *et al*. 2010 (23). Further more, it was observed that there was no significant difference in encapsulation efficiency with different concentrations of calcium carbonate and CaCl_2_ used as gas forming and cross linking agent, respectively. The drug content of COB formulation was found to be in the range of 61 ± 0.4 to 82 ± 0.6% w/w, HOB formulation was found to be in the range of 48 ± 0.6 to 69 ± 0.8 % w/w. No significant (p < 0.05) effect was observed with CaCO_3_ and CaCl_2_ on the drug content of beads.

**Table 3:** Characterization of prepared formulation of polymeric blended gellan gum beads

**Formulation**		**Diameter** **(mm)** ^a,b,c^		**Floating ability (%)** ^a,c^		**Encapsulation** **Efficiency** **(%w/w)**^a, c^		**Drug content % ** ^a,d ,c^	
**COB**	**HOB**	**COB**	**HOB**	**COB**	**HOB**	**COB**	**HOB**	**COB**	**HOB**
K_1_	S_1_	1.85 ± 1.3	1.3 4± 1.4	85 ± 0.4	76 ± 0.4	82 ± 1.6	75 ±1.3	68 ± 0.5	58 ± 0.4
K_2_	S_2_	1.90 ± 1.5	1.42 ± 1.5	82 ± 0.6	73 ± 0.6	86 ± 1.4	73 ±1.5	63 ± 0.8	51 ± 0.5
K_3_	S_3_	2.17 ± 1.2	1.48 ± 1.6	77 ± 0.4	68 ± 0.4	79 ± 1.7	74 ±1.5	61 ± 0.4	48 ± 0.6
K_4_	S_4_	2.28 ± 1.3	1.39 ± 1.4	82 ± 0.2	70 ± 0.5	96 ± 1.8	84 ± 1.4	82 ± 0.6	67 ± 0.4
K_5_	S_5_	2.08 ± 1.8	1.70 ± 1.5	74 ± 0.3	79 ± 0.6	94 ± 1.4	88 ± 1.6	73 ± 0.9	69 ± 0.8
K_6_	S_6_	2.20 ± 1.6	1.62 ± 1.6	66 ± 0.4	63 ± 0.5	85 ± 1.5	79 ± 1.4	64 ± 0.7	66 ± 0.5
K_7_	S_7_	1.88 ± 1.2	1.47 ± 1.4	61 ± 0.4	60 ± 0.4	86 ± 1.4	82 ± 1.8	68 ± 0.3	64 ± 0.6
K_8_	S_8_	2.03 ± 1.3	1.60 ± 1.2	69 ± 0.4	63 ± 0.5	85 ± 1.8	84 ± 1.5	64 ± 0.6	62 ± 0.3

COB = gellan: carbopol blended formulation. HOB = gellen : HPMC blended formulation. a: Mean ± SD (n = 3). b: n = 20. c: n = 3. d: Drug content in each 100 mg of bead

*In-vitro drug release*

**Figure 3:**
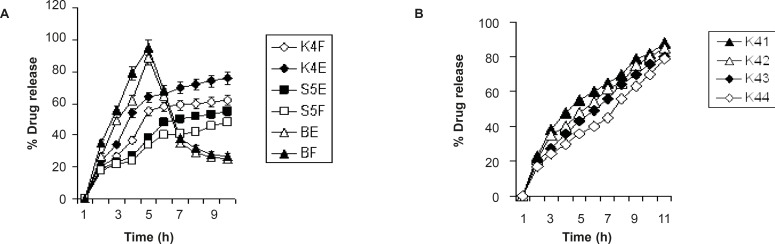
Comparative drug release profile: (A) drug release in fasted and fed state; (B) drug release in fed state.

*In-vitro *release study of Amoxicillin gel beads was carried out in the fasted state (SGF solution, pH = 1.2) and fed state (phthalate buffer, pH = 3.4) for a period of 8 h. The results indicate that 62 ± 1.4% of the pure drug (batch BE) was dissolved within 2 h in fasting state (pH of 1.2) and in the fed state (batch BF, pH of 3.4) and the release was 79 ± 1.2 %. After 8 h, drug release from batch K_4_E and batch K_4_F was 76.0 ± 2.1% and 62.0 ± 2.2% (fasting state and fed state) in the SGF and phthalate buffer solution (Figure 3a), respectively. Drug release from the optimized formulations K4 followed the Higuchi (R_2_ = 0.984) and Peppas models (R_2_ = 0.946, n= 0.36) and suggested a diffusion based mechanism of the drug release as the diffusion exponent values were less than 0.45 (23).

Batch K_4_ of the formulation COB was selected for optimization through coating with ethyl cellulose in order to modify the drug release pattern (24). However, the dissolution profiles of all EC-coated beads were best fitted to the zero-order kinetic model (Figure 3b). The batch K_41_ which showed the highest release (Table 2) of amoxicillin (88 ± 1.3%) was regarded as pH sensitive controlled release formulation of amoxicillin.

*In-vitro growth inhibition studies*

**Figure 4:**
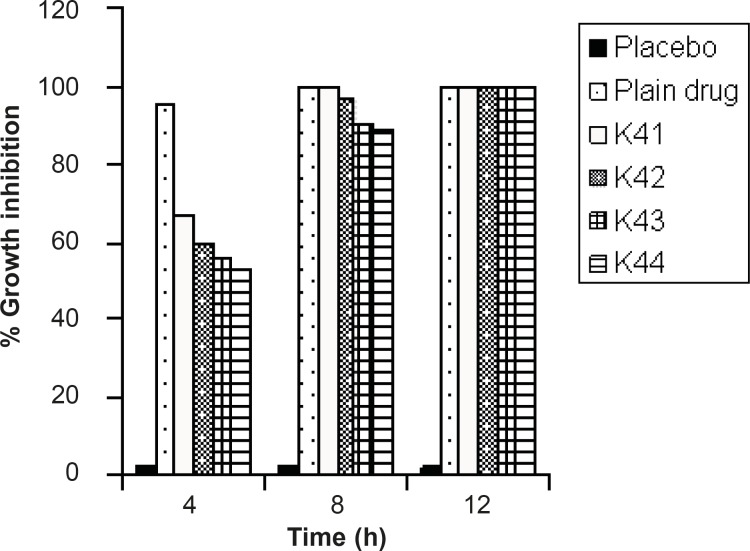
Percentage growth inhibitions of formulations

The antimicrobial efficacy of the prepared drug-loaded, placebo (drug-free) beads and plain drug was investigated at various time intervals in the isolated *H.pylori *bacterial strain up to 12 h and the results are shown in Figure 3. The EC-coated formulation (K_41,_ K_42,_ K_43,_ K_44_), the placebo and the plain amoxicillin were selected for *in-vitro *growth inhibition studies. Culture tube of *H. pylori *containing placebo bead did not show significant percent growth inhibition (2.3 ± 1.2 %) and the percentage of growth inhibition sequence in the coated formulation was varied (K_41_ (70 ± 1.2 % ) >K_42_ (67 ± 1.4 % ) >K_43_ (62 ± 1.3 % ) > K_44 _(60 ± 1.2 %)) whereas, the plain amoxicillin inhibited hundred percent *H. pylori *growth at 4 h of the studies (Figure 4). Continuing the incubation of *H. pylori *up to 8 h, the optimized formulation (batch k_14_) was eradicated hundred percent growth of the *H. pylori *and through further incubation for 12 h, all the optimized coated batches showed complete termination of the microbial infection.

This was due to the controlled delivery of amoxicillin from the formulation and the long time exposure of the microorganism in the MIC state which resulted in complete clearance of *H. pylori*. EC-coated formulation (batch S_41)_ was more effective in term of the microbial eradication, abolished all the mechanisms of the bacterial survival *in-vivo *and may provide targeted eradication of *H. pylori *cell lines.

## Conclusions

Design formulation contains optimized ratio of the blended hydrophilic polymer, allowing the initially penetration of the drug into the unique niche of the gastric mucosa. The preferential accumulation of the formulation in the stomach mucosa may be very useful for targeting the antibiotics at the site of infection and also possibly for *H. pylori *eradication. Thus, from the result, we concluded that the optimized batch K_41_ appears to have promising potential for opening up possibility for targeting antibiotics like amoxicillin at the site of infection and also for *H pylori *eradication.
